# Death of Dr. Waddel of Toledo

**Published:** 1879-05

**Authors:** 


### Death of Doctor Thomas Waddel of Toledo, Ohio.

On Sunday evening, March 9th, Dr. THOMAS WADDEL died at his late residence, No. 649 Huron Street. Cut down in the midst of an active, useful life, the bereavement is made doubly so by its suddenness; scarcely was it known that he was sick before his death occurred. On Saturday morning he visited his patients as usual; on Sunday night he passed beyond the cares of earth to reap the reward of one who had well borne his part in the struggles and trials of life.

On Friday he had a chill but was able to attend to a case of labor in the afternoon, and though suffering with fever he terminated the protracted case with forceps. Saturday morning he made his calls but at noon was obliged to take to his bed, which he never left. * * * * * * * * * * * *

We can give but a brief outline of his life and work. Those who knew him best feel that in his death they have sustained a loss which cannot be repaired and that a life full of promise, of usefulness and brilliant success has been brought to a premature close. At the time of his death his age was 35 years, 4 months and 26 days, having been born in Canada on the 13th of October, 1843. He came to the States in 1865, entering the Medical University of Buffalo. In 1870 he attended the lectures in the University of Wooster, Cleveland, graduating with the highest honors at that institution in 1871. He went to Toledo near the close of 1873, and during his residence there took rank among the most prominent physicians of the city, and met with eminent success in his profession. He was lecturer on Gynecology, in the Toledo School of Medicine; was a member of the medical staff of the St. Vincent Hospital; a member of the Board of Health, and also of the American, State, Northwestern Ohio and Toledo Medical Associations.—*Toledo Med. & Surg. Journal.*

